# A Remarkable Case of Poisoning

**Published:** 1880-10-20

**Authors:** A. F. F. Kerstan

**Affiliations:** Atlanta, Ga.


### A REMARKABLE CASE OF POISONING.

[Translated from "Medicinische Newkeiten fur Practische Arzte Erlangen." January 1st 1880.]

BY A. F. F. KERSTAN, M.D., ATLANTA, GA.

In the Daily News a remarkable case of poisoning is reported, which occurred in Saxony. A young engineer who was employed since November of the past year in the machine manufactory of his father to make drawings, sank suddenly down on his workingtable. From the results of a *post-mortem* examination, the family physician suspected poisoning. This suspicion was confirmed by physicians who made a second *post-mortem* examination. After that an examination of various parts of the corpse, at the Central Laboratory in Dresden, proved the following result:

The œsophagus contained no trace of arsenic; the stomach and its contents only the most minute traces of it; but it was very clearly possible to detect its presence in the small intestines, liver, kidneys, lungs, heart and brain. From the abundance of arsenic in the brain was necessarily inferred a very carefully contrived poisoning, which possibly might be considered only in a secondary degree as the cause of death. This conclusion was justified by still other facts. The circumstance that the young man had lived in very happy domestic relations excluded the probability of an intentional suicide or of a murder by others. The physical state of the man had been disturbed of late, and on the last day of his life he had complained of severe headache. Hence his surviving kindred fixed their attention upon his former manner of labor, and consequently they sent to the Central Laboratory, in Dresden, a great many objects for examination—among them various water-paints which the deceased had used in the latter part of his life, as was well known. The chemical examination of these water-paints proved that tusch, gamboge, carmine, blue, red kosin-ink and neutral-ink were entirely free of arsenic. A specimen of sepia contained 1.08 per cent.; a specimen of terre de sienne, with the stamp "F. M. Paillard," 3.14 per cent.; a red-brown paint even 3.15 per cent. of arsenious acid. Sepia with the firm's stamp "Chenal, Paris," and burnt and unburnt terre de sienne with the term "technical paint," were proven to contain arsenic. These paints the young man had formerly used in the Polytechnical School. And he had the habit of bringing the paint-saturated brush to his lips and pointing it between them. It was conceded in this way there was a gradual introduction of the arsenic-containing paint by means of the saliva, that finally brought on the poisoning.

